# Utility of Pelvic Floor Therapy and Behavioral Interventions in the Neurogenic Lower Urinary Tract Dysfunction (NLUTD) Population

**DOI:** 10.1002/nau.70249

**Published:** 2026-02-23

**Authors:** Sara M. Lenherr

**Affiliations:** ^1^ Department of Surgery, Division of Urology University of Utah Salt Lake City Utah USA

**Keywords:** behavioral therapy, multiple sclerosis, neurogenic lower urinary tract dysfunction, pelvic floor muscle therapy, spinal cord injury

## Abstract

**Aims:**

Neurogenic lower urinary tract dysfunction (NLUTD) describes a wide range of symptoms depending on neurologic diagnosis and the resulting bladder and sphincter dysfunction. This narrative review is designed to address the question regarding which NLUTD population benefits most from pelvic floor therapy and behavioral interventions, and how to deliver these therapies.

**Results:**

Most NLUTD treatment guidelines encourage least‐invasive, low‐risk therapies like behavioral therapy (BT) and pelvic floor muscle therapy (PFMT)/pelvic floor physical therapy (PFPT), but they are often underutilized because of a multitude of reasons. Studies show that PFMT along with electrostimulation or biofeedback to facilitate understanding of the pelvic floor activity, may offer significant improvement in urinary symptoms and quality of life in certain NLUTD populations.

**Conclusions:**

Education, adherence support, and supervised exerciseappear to enhance outcomes. Finally, evidence suggests that telerehabilitation‐based PFMT is feasible, effective and associated with higher adherence and satisfaction compared with home exercise alone.

**Abbreviations**
AIS: American Spinal Injury Association Impairment Scale
BT: Behavioral Therapy
EDSS: Expanded Disability Status Scale
EMG: Electromyography
HRQoL/QoL: Health‐Related Quality of Life
ICIQ‐SF: International Consultation on Incontinence Questionnaire–Short Form
MMC: Myelomeningocele
MS: Multiple Sclerosis
MSQoL‐54: Multiple Sclerosis Quality of Life–54
NLUTD: Neurogenic Lower Urinary Tract Dysfunction
NMES: Neuromuscular Electrical Stimulation
OABQ‐SF: Overactive Bladder Questionnaire–Short Form
PD: Parkinson's Disease
PFMT: Pelvic Floor Muscle Therapy
PFPT: Pelvic Floor Physical Therapy
SCI: Spinal Cord Injury
SF‐36: 36‐Item Short Form Health Survey
UI: Urinary Incontinence

## 1 Introduction

Care for patients with NLUTD requires a balance of interventions and shared‐decision making. Often, patients present with urinary storage and emptying symptoms that are exacerbated by mobility issues and/or cognitive impairment. For example, providers need to consider the risk of a person with Parkinson's Disease (PD) falling when urgently running to the bathroom. Or the time‐consuming pelvic floor physical therapy (PFPT) visits for a young working woman with multiple sclerosis (MS). While we intend to do the best for our patients, providers often overlook the possible benefits of minimally invasive therapies in an attempt to offer more confident and discrete treatments. Our hesitation to encourage PFPT or BT to patients with NLUTD is not unfounded. Because of the rigor needed to demonstrate benefit of these minimally invasive therapies, most studies have traditionally excluded persons with neurologic dysfunction. These studies not only were limited to able bodied populations, but also to women that were more likely to experience lower urinary tract dysfunction from childbirth. Extrapolating results from interventions on predominantly female populations is reasonable in MS which primarily affects women, but spinal cord injury (SCI) and PD primarily effects men. It is only in the last 20 years that pockets of experts have tried to design and demonstrate utility of these interventions for both men and women with NLUTD.

In general, the interventions discussed in this review inherently require patients have some degree of voluntary motor function and intact sensation, although level of disability can be surprisingly quite high and still have some benefit. Interventions that are passive such as neuromodulation are outside the scope of this review, but neurostimulation and biofeedback are often used as adjuncts for PFMT and BT. This narrative review will present the most current data regarding the utility of pelvic floor therapy and behavioral interventions in the most pertinent categories of patients that experience NLUTD. Relevant studies were identified through targeted review of peer‐reviewed journals, with emphasis on seminal and influential publications that shaped the evolution of this narrow, poorly understood field.

## 2 Intervention Description: BT, PFMT, and PFPT

Interventions should be offered after an initial baseline evaluation, including physical examination, cognitive assessment, and administration of bladder‐related symptom questionnaires. Shared‐decision making about goals of care and ability to participate should be balanced with the potential benefits of the therapy.

### 2.1 Behavioral Therapy (BT)

Behavioral therapy is the most basic intervention along with patient education about bladder function. One of the most comprehensive descriptions of behavioral therapy was described in the ESTEEM trial (Effects of Surgical Treatment Enhanced with Exercise for Mixed Urinary Incontinence Trial) [1]. They developed a protocol based on multidisciplinary BT instructional and motor learning principles. Fundamental elements of BT include

− education about lower urinary tract function
− pelvic floor anatomy and function
− voiding habits
− instruction on timed voiding with incremental increases
− urge suppression
− normalization of fluid intake
− dietary bladder irritant information
− constipation management

The patient's level of cognitive engagement is a critical element of BT. Occupational therapists can also recommend adaptive home equipment to simplify getting to the bathroom and proper sitting on the toilet to void.

### 2.2 Pelvic Floor Muscle Therapy (PFMT)

For clarity, this review will refer to pelvic floor muscle therapy (PFMT, sometimes called pelvic floor muscle exercises, PFME) as activities to addresses the function of pelvic floor muscles. This activity is performed independently by the patient after being provided written instructions, verbal coaching and/or physical cueing provided during examination [2]. Surprisingly, the exact mechanisms by which PFMT helps storage symptoms is not understood. The act of pelvic floor muscle contraction is postulated to (1) strengthen voluntary baseline muscle tone, (2) promote maximized awareness to contract the pelvic floor, and (3) strengthen the core muscles which co‐engages the pelvic floor [3]. This combination is thought to cause perineal‐detrusor reflex activation, resulting in decreased bladder urgency [4, 5].

### 2.3 Pelvic Floor Physical Therapy (PFPT)

Pelvic floor physical therapy (PFPT) on the other hand, is typically conducted with assistance or a specialized pelvic floor physiotherapist. This includes both the internal and external assessment of these muscles and associated musculature, followed by internal and external manual therapy, a program of education and guided exercises that address muscle tone, strength, coordination, and overall function including pain. Often, the initial visit with most pelvic floor physiotherapist will assess goals of care and lower urinary tract symptoms. Once a rapport is established, a physical examination with active feedback will follow at the same or subsequent visits. Most effective PFPT is accomplished with the use of digital examination and manual therapy, rather than just external palpation. PFPT exercises can be performed in the supine or sitting positions, with progression to more complex positions. Repetition of the exercises is done with monitoring for fatigue. Certified PFPT providers are very skilled at their assessments, provide compassionate care and can create specialized treatment protocols for their patients.

### 2.4 Biofeedback

Biofeedback can be used as an adjuvant for PFPT treatments to provide the patient and provider information during PFMT exercises. The biofeedback information is obtained with EMG electrodes to measure bioelectrical activity of the pelvic floor muscles. This is a measurement of muscle activation, not muscle strength or pressure. Vaginal, anal and/or surface electrodes can be used to show the contraction and relaxation phases of the pelvic floor muscles. The combination of visual and auditory feedback during contraction and relaxation is used to coach patients on muscle location, dyssynergy and fatigue. This enables the patient to more accurately understand how to use their pelvic floor muscles correctly. As awareness improves, the biofeedback can be reduced or discontinued. Pressure biofeedback with vaginal or rectal sensors is an alternative, measuring changes in pressure. However, pressure biofeedback not sensitive to only pelvic floor activity and can be prone to confounding from Valsalva and abdominal recruitment.

### 2.5 Augmented Therapies

“Electrostimulation” or more precisely, neuromuscular stimulation (NMES) can be used to augment PFPT. This is accomplished with the placement of an intravaginal/intrarectal probe that applies an electrical current to depolarize the peripheral motor nerves in the pelvic floor, causing an involuntary muscle contraction. The contraction relies on intact lower motor neuron pathways. This can be a valuable adjunct when the patient is unable to voluntarily contract the pelvic floor, but it should not be used in the setting of pelvic pain or dyssynergic voiding.

## 3 Patient Populations

### 3.1 Multiple Sclerosis (MS)

Lower urinary tract symptoms (LUTS) represent the most predominant and disabling non‐motor manifestations of MS, present in an estimated 50%–90% of patients. Table 1 presents the research to date chronologically by research group, as often the same population or study design is followed over time. PFMT often combined with BT, biofeedback, or electrical stimulation, has been widely studied across several small, single‐center groups. It is worth noting that across the included trials, the MS patients had moderate disability as quantified by the Expanded Disability Status Scale (EDSS) score ≤ 6.5. Specifically, they were able to walk but do not require two walking aids continuously. However, one study did include those with EDSS score ≤ 8, indicating more severe disability [14].

**TABLE 1:** Summary of studies on PFMT in patients with multiple sclerosis.

Author	Year	N (% female)	Arm 1	Arm 2	Arm 3	Length	Primary outcome	Secondary outcome
Vahtera [6]	1997	80 (62%)	PFMT with electrical stimulation	No treatment	—	26 weeks	Improved storage symptoms in treatment group compared to control	Improved QoL with PFMT
McClurg [7]	2006	30 (100%)	PFMT	PFMT with biofeedback	PFMT, biofeedback, & NMES	24 weeks	Significant improvement in UI in arm 1 and arm 3 compared to arm 1 (*p* < 0.01)	Improvement in urinary symptoms & QoL
McClurg [8]	2008	74 (77%)	PFMT, EMG biofeedback, sham NMES	PFMT, EMG biofeedback, active NMES	—	24 weeks	Significant improvement in UI with the addition of NMES (*p* < 0.01)	Significantly improved pad weights, PVR and pelvic floor muscle strength and QoL
Lúcio [9]	2010	27 (100%)	PFMT	Sham	—	12 weeks	Improved storage and voiding symptoms compared to sham	Reduced incontinence pad weights, improved muscle power, endurance resistance & contractions
Lúcio [10]	2011	35 (100%)	PFMT	Sham	—	12 weeks	Improved storage and voiding symptoms compared to sham	Improved QoL with PFMT
Lúcio [11]	2016	30 (100%)	PFMT with EMG and sham NMES	PFMT with EMG and NMES	PFMT with EMG and TTNS	12 weeks	No difference between groups in incontinence pad weights	PFMT with NMES was more effective in decreasing pelvic floor tone
Ferreira [12]	2016	24 (100%)	PFMT	PFMT with electrotherapy	—	26 weeks	PFMT with electrotherapy improved OAB symptoms (*p* = 0.04) and perineal contraction (*p* < 0.01) compared to PFMT alone	
Ferreira [13]	2019	30 (100%)	PFMT	PFMT with NMES	—	6 months	Both PFMT with or without NMES improved QoL and OAB symptoms.	PFMT with NMES was more effective in increasing perineal muscle contraction
Perez [2]	2020	40 (55%)	“Unguided” PFMT	“Guided” PFMT with physiotherapist	—	12 weeks	No significant difference in UI (*p* = 0.21)	Physiotherapist arm had improved QoL, urinary symptoms
Yavas [14]	2023	45 (80%)	Tele‐PFMT	Home‐PFMT	control	8 weeks	Both Tele‐ and Home‐PFMT improved UI and pad use over control on bladder diary	No differences between Tele‐ and Home‐ PFMT QoL or UI questionnaires

Urinary incontinence outcomes consistently were shown to improve across multiple studies with PFMT. PFMT reliably reduced urinary leakage episodes, pad weight, and pad usage compared with sham or no‐treatment controls [7, 8, 9]. These studies demonstrated statistically and clinically meaningful reductions in leakage frequency following 8–24 weeks of training.

The addition of neuromuscular electrical stimulation (NMES) frequently enhanced outcomes. Both McClurg et al. [8] and Lúcio et al. [11] showed greater reductions in incontinence episodes when NMES was added to PFMT compared with PFMT alone or PFMT with placebo stimulation. For the McClurg study, the active NMES therapy resulted in an 85% reduction in incontinence episodes compared with 47% in the placebo NMES group, with statistically superior outcomes across multiple objective and patient‐reported measures. These findings strongly support NMES as a meaningful adjunct to PFMT and BT in MS‐related NLUTD.

Urgency, frequency, nocturia, and urge urinary incontinence improved significantly following PFMT‐based interventions [6, 9, 10, 12, 13]. Improvements were documented using bladder diaries and validated instruments.

Nearly all trials that formally assessed pelvic floor muscle performance reported significant gains in muscle strength, endurance, and rapid contraction ability following PFMT [8, 9, 11, 12, 13]. NMES appeared particularly effective in improving muscle tone regulation and relaxation capacity [8, 11, 13], which may be relevant in MS‐related dyssynergia.

Improvements in urinary symptoms translated into meaningful gains in health‐related quality of life (HRQoL), as measured by SF‐36, Qualiveen, ICIQ‐SF, MSQoL‐54, and disease‐specific instruments [2, 6, 7, 8, 10, 13, 14]. Reductions in anxiety and depressive symptoms were also reported, particularly in studies incorporating supervised therapy [12].

One recent study addresses a practical and clinically important question: does physiotherapist guidance matter? [2] This group conducted a randomized trial comparing unguided‐ versus guided‐PFMT in individuals with relapsing–remitting MS, EDSS score ≤ 6.5. The unguided‐PTMT group was followed home training program provided by verbal and written instruction manual. No physical exam or coaching was given. The home training manual also included behavioral therapy information about typical incontinence triggers. The guided‐PFMT group received the above instruction along with weekly 30‐min physiotherapist appointments where they underwent internal (intrarectal or intravaginal) coaching. If the participant declined the internal examination, they were able to receive external palpation assistance with palpation of the central fibrous nucleus of the perineum. All treatments were expected to be done three times daily. Interestingly, both groups showed similar reductions in leakage episodes as documented on a 3‐day bladder diary at 2‐ and 3‐months follow‐up. However, the guided‐PFMT group also demonstrated greater improvements in urinary symptom severity as determined by the OABQ‐SF [15], and quality of life, with trends toward better adherence. These findings suggest that while PFMT can be effective as a home program, professional supervision enhances symptom control, and broader patient‐centered outcomes.

Finally, in an era of telemedicine, Yavas, et al. evaluated the feasibility of Tele‐PFMT versus Home‐PFMT in patients with MS and EDSS score ≤ 8 and without any recent medication changes or MS‐related decline [14]. All participants first met with a physiotherapist in person for their first visit for PFMT instruction and were provided a training brochure with exercises and progression information. They were instructed to perform the exercises three times daily for 8‐weeks. Tele‐PFMT was conducted in real time via videoconference twice weekly. The participant was visible on the screen and did not remove any clothing. The physiotherapist demonstrated and verbally coached the patient, changing positions and number of repetitions with time. The control group received standard MS and bladder care, including medications. Engagement in the Tele‐PFMT was proven to be feasible and effective. Participants in both the Tele‐PFMT and Home‐PFMT groups had a decrease in leakage episodes and pad usage based on 3‐day bladder diary, and both were improved over the control arm.

Overall, this collection of studies supports the fact that PFMT, ideally with a physiotherapist, has the potential to be an important, low‐risk adjunct to manage NLUTD with MS. Unfortunately, the quantitative impact of this intervention is unknown because of small sample sizes and limited generalizability (most studies are single center). Additionally, recent systematic reviews and a meta‐analysis identify possible bias and limitations of this collection of studies [16, 17, 18].

### 3.2 Spinal Cord Injury (SCI) and Myelomeningocele (MMC)

The utility of BT and PFMT in those with SCI might often be overlooked, however incomplete SCI (AIS‐B, C, D & E) and mild MMC are often ambulatory. They frequently have voluntary control over their pelvic floor and some bladder sensation. There are very few publications on PFMT and SCI or MMC. An investigator‐blinded parallel‐group randomized clinical trial in women with incomplete SCI or MMC and UI was performed with two intervention groups: (1) PFMT versus (2) PFMT with intravaginal NMES [19]. Only four out of 26 women had normal sensation by pin prick and light touch in the S3 and S4–S5 dermatomes. At 12‐ and 24‐weeks follow‐up, the PFMT group had a significant improvement in UI (25% reduction of UI episodes), but no change in the PFMT with NMES group (3% reduction of UI episodes). It was postulated that the NMES was too complex for users while performing the PFMT because of their impaired hand function trying to hold the NMES device. Locomotor training is also postulated to be highly beneficial to bladder function after SCI, but the results have been mixed [20]. But, because of some improvements in bladder awareness [21, 22, 23, 24] and urine leakage [22, 23, 24], more clinical trials are ongoing to further explore this intervention. The underlying theories about how locomotor training improves bladder function, are thought provoking and might really expand our knowledge of neuroplasticity. However, many of these studies are conducted with more complex neuromodulation and transcranial magnetic stimulation [20].

### 3.3 Parkinson's Disease (PD)

Parkinson's disease most commonly presents with overactive bladder symptoms followed by bladder outlet obstruction. Three studies have evaluated PFMT and BT in patients with PD [25, 26, 27]. Together these studies show improvements in UI episodes and HRQoL on bladder diary and standardized questionnaires, respectively. Given the favorable side effect profile of behavioral therapy compared to other treatment options such as drug therapy or other interventions, it is reasonable for clinicians to offer behavioral therapy as a first‐line treatment option to persons with Parkinson's disease.

### 3.4 Stroke & Traumatic Brain Injury

Stroke can variably impact control of voiding with a reported incidence of 28%–79% [28]. Often these patients have abnormal sensation of bladder fullness and urgency sensation with small volumes of urine in the bladder. There is a concomitant issue with walking as a result of the stroke [29]. Behavioral toileting strategies, toileting schedules/prompted voiding, pelvic‐floor muscle training (PFMT) are helpful in this population, perhaps more so women than men [28, 29, 30, 31].

## 4 Discussion

The heterogenetic nature of NLUTD in patients with retained motor and sensory function might limit the perceived benefit of BT and PFMT. Physician knowledge about these interventions and time resources also limit adoption. Providers might offer these interventions as an initial therapeutic trial, rather than a confident endorsement. From the patient perspective, these interventions can be costly and time‐consuming, further discouraging adoption. But, studies presented here might be encouraging regarding telehealth care for PFMT. Additional barriers of care include insurance coverage (if covered at all), co‐pays, time, travel distance, training and the awkward nature of the intervention. Persistence with therapies might be limited because of perceived lack of benefit, frustration, and the desire to move to more advanced therapies. Monitoring adherence is not something that most providers are often able to do outside of a clinical trial. However, there is enough evidence to encourage patients to seek a knowledgeable physical therapist and personalized program.

Despite these limitations, BT and PFMT should be offered to most patients presenting with any lower urinary tract symptoms. In general, PFMT is cost‐effective, effective, and accessible treatment once a physiotherapist is identified. Professional physical therapy society registries can facilitate finding local providers. PFMT may be synergistic with overactive bladder medications, offering a more significant overall incontinence improvement. It can also be an effective treatment of coexisting stress incontinence, and additional non‐urinary improvements in bowel and sexual function, further justifying the added time and potential expense associated with PFMT.

This field needs studies to further understand how pelvic floor activity impacts bladder function. In the meantime, exoskeleton gait training is being used to target NLUTD in SCI patients [32] and other potential interventions include reflexology and acupuncture [33, 34].

## 5 Conclusions

BT and PFMT with or without NMES should be considered for many patients with NLUTD. The evidence is most robust in MS patients, but the literature is developing for those with incomplete SCI, Parkinson's Disease and other related populations. Multidisciplinary care with physiatrists, physiotherapists, occupational therapists, nurses, and urologists is likely key to successful therapy. Fortunately, more patients are seeking care regarding NLUTD. Urologists have an opportunity to increase their knowledge about the benefits of BT and PFMT for patients experiencing NLUTD.

## Ethics Statement

The author has nothing to report.

## Author Contributions

S. Lenherr is responsible for conception, data collection, writing and editing.

## Funding

The author has nothing to report.

## Conflicts of Interest

The author declares no conflicts of interest.

## Data Availability

All data are available upon request.
